# Patient and Practitioner Perspectives on the Definition and Measurement of Therapeutic Empathy: Qualitative Study

**DOI:** 10.2196/71610

**Published:** 2025-07-31

**Authors:** Amber Bennett-Weston, Jeremy Howick

**Affiliations:** 1 Stoneygate Centre for Empathic Healthcare, Leicester Medical School, University of Leicester, George Davies Centre, Lancaster Road, Leicester, LE1 7HA, United Kingdom, 44 7398858819

**Keywords:** therapeutic empathy, empathy, patient-practitioner communication, definition, measurement

## Abstract

**Background:**

Most definitions of therapeutic empathy are based on practitioners’ perspectives, and few account for patients’ views. Therefore, we do not understand what therapeutic empathy means to patients. Given that therapeutic empathy involves a relationship between patients and practitioners, the underrepresentation of the patient voice threatens to undermine the validity of therapeutic empathy definitions and subsequently, how the concept is measured, taught, and practiced.

**Objective:**

The aim of the study is to explore the perspectives of patients and practitioners on the definition of therapeutic empathy and how it should therefore be measured.

**Methods:**

A qualitative study, underpinned by a social constructivist stance, was conducted. Patients and practitioners were purposively sampled from a medical school and a school of health care to represent a diversity of lived experiences and health care professions. In-depth, semistructured interviews were undertaken, and the data were analyzed using reflexive thematic analysis. Data collection ceased upon reaching meaning saturation.

**Results:**

In total, 16 participants (8 patients and 8 practitioners) were interviewed in June and July 2024. Reflexive thematic analysis generated three overarching themes that synthesize the views of patients and practitioners on therapeutic empathy and how it should be measured: (1) therapeutic empathy involves the practitioner showing the patient (that they are interested in the patient as a person, that they are actively listening, that they understand, that they are emotionally engaged, and that they are responding to their needs), (2) context matters (eg, the clinical scenario, time, and the patient), and (3) short, simple scales are a pragmatic approach to measurement.

**Conclusions:**

Patients and practitioners have similar views about what empathy is and define therapeutic empathy as involving the practitioner demonstrating specific attitudes and behaviors to their patients. These attitudes and behaviors should be included in interventions to enhance therapeutic empathy and in measures of the concept. However, contextual factors may influence the expression of therapeutic empathy in practice. The findings highlight the need for, and can inform the development of, a short therapeutic empathy scale that allows the comparison of scores between patients, practitioners, students, and observers.

## Introduction

### Rationale

Over recent decades, therapeutic empathy (sometimes called “clinical empathy”) has become a central tenet of health care practice, research, and education [1-3]. This is unsurprising, given the growing body of research demonstrating its benefits for patients (reduced pain and anxiety and improved satisfaction) [4,5] and practitioners (reduced burnout and improved job satisfaction) [6,7]. Alongside the increasing interest in therapeutic empathy, there has been ongoing controversy surrounding its definition [8]. In particular, authors have debated whether therapeutic empathy is a cognitive (requiring understanding) [9] or affective (requiring feeling) concept [10] or indeed whether it includes both cognitive and affective components [1,11]. A recent review of 39 definitions found that definitions of therapeutic empathy share 6 common components: *exploring* and *understanding* the patient’s perspective, reaching a *shared understanding* with the patient, *feeling* in response to understanding, and taking *therapeutic action*, all while *maintaining boundaries*—both personal and professional [11].

This review highlighted that most therapeutic empathy definitions have been developed by and for practitioners and rarely account for patients’ perspectives [11]. To wit, a recent scoping review [12] identified only 4 studies [13-17] that explored patients’ experiences of therapeutic empathy. Even these studies were limited to specific patient groups (patients with cancer [13,15,17] or comorbid pain and depression [16]), so the scope of patient representation was limited. A related problem is the near absence of research exploring how patients’ perspectives on therapeutic empathy align with practitioners’ perspectives and the similarities between them [13,17]. Those who do, position empathy as paying empathic attention to the patient, emotional engagement, and putting oneself in the patient’s shoes [13,17]. One study highlighted that the views of patients and practitioners on therapeutic empathy differed greatly [17]. However, in the same way that these studies are limited to particular patient groups, they are limited to specific practitioner populations, including nurses [13] and oncologists [17].

The paucity of patients’ perspectives on the definition of therapeutic empathy is problematic for 2 reasons. First, there could be a mismatch between what practitioners believe to be empathic care and what patients perceive as empathic care. This, in turn, is likely to lead to worse patient outcomes [4,5]. Indeed, teaching therapeutic empathy currently focuses on communication skills training, such as active listening and perspective-taking [2]. However, it is unclear whether these educational interventions include all of the necessary elements to successfully train practitioners to be perceived as empathic by their patients [15]. This could explain the great variation in the extent to which patients rate practitioners’ empathy levels [18] and the discrepancy between practitioners' self-ratings of empathy and patients' ratings of practitioners' empathy [19-21]. Second, and relatedly, failure to adequately consider patient views threatens to violate standards of good practice for patient involvement in the development and delivery of health care services and interventions [22,23].

Underrepresentation of the patient voice in the definition of therapeutic empathy also creates problems for research, particularly with regard to measurement of the concept. Of the many measures purporting to assess therapeutic empathy [24-31], few are directly informed by patients’ perspectives [15,32]. This is problematic because the measurement of any concept should account for the lived experiences of all stakeholders [33,34]. Measures that are not informed by all stakeholders risk having poor content validity (the adequacy with which a measure assesses the intended concept) and poor user-friendliness [33,34]. Indeed, widely used measures of therapeutic empathy, such as the Jefferson Scale of Empathy [31], have been criticized for excluding important components of the concept (such as emotion or therapeutic action) [35] and for being long and unwieldy [24-31,36]. These problems, in turn, threaten the integrity of the conclusions drawn from empirical studies measuring therapeutic empathy and its relationship to other variables.

As such, there is a need for research exploring the views of patients and practitioners on the definition and measurement of therapeutic empathy to ensure that the teaching, practice, and research of the concept are grounded in the lived experience of key stakeholders. This is especially important, given the relational nature of the concept [11] and the growing interest in it in health care contexts [1].

### Research Aim

This study aimed to explore the perspectives of patients and practitioners on the definition of therapeutic empathy and how it should therefore be measured.

## Methods

### Design

We conducted an exploratory qualitative study underpinned by a social constructivist philosophical stance. From this perspective, meaning is actively constructed, tested, and modified by individuals through social interaction [37]. This stance was selected, as therapeutic empathy is described as being interactive [11].

### Ethical Considerations

Ethics approval for this study was received from the University of Leicester’s Research Ethics Committee (reference: 0375) in May 2024. All participants provided informed consent to take part in the study. Patient participants were remunerated in line with the University of Leicester’s Patient and Carer Group hourly pay rate.

### Sampling and Recruitment

We recruited patients from a patient and carer group who are involved across a medical school and school of health care and practitioners from the same 2 schools. This provided us with access to patients who represent diverse lived experiences of health and care and practitioners from a variety of health care professions. This was important, given that previous research has been limited to specific patient or practitioner groups [13,15,16]. We purposively sampled participants for maximum variation [38], including patients of different sexes, ages, and ethnicities, with diverse lived experiences, and practitioners of different sexes, ages, and ethnicities, from different professions. Purposefully sampled participants were recruited via their university email following ethics approval and permission from the relevant gatekeeper: the head of each school or the patient and carer group chair.

### Patient Involvement

A patient advisory board was involved in the study design and the analysis. One researcher (AB-W) met with the board, which was comprised of 3 patient representatives, at the inception of the study and during analysis. The board helped to shape the topic guides to ensure accessibility for patient participants and offered insights into the interpretation of the data. Specifically, they highlighted the behavioral nature of participants’ definitions of therapeutic empathy.

### Data Collection

As a social constructivist study, it was important that the data were constructed through social interaction [37]. Accordingly, we conducted semistructured interviews aided by topic guides (Multimedia Appendix 1). The interviews explored, in depth, participants’ own meanings and experiences of therapeutic empathy, along with their views on how it should be measured. To accommodate participants’ needs, we provided the option of conducting the interviews in person or digitally. Sampling, data collection, and analysis were iterative and concurrent; we ceased data collection when we had reached meaning saturation [39]. This is the point at which we had developed an in-depth understanding of the complexities and nuances of the concepts generated through data collection [39].

### Data Analysis

Interviews were audio recorded, transcribed verbatim, and analyzed in NVivo (Lumivero). We analyzed the data using reflexive thematic analysis, a 6-phase approach to identify patterns of meaning in qualitative data [40]. Our approach to analysis was predominantly inductive to remain grounded in participants’ accounts [40]. In phase 1 (familiarization), 1 author (AB-W) read each transcript several times. In phase 2 (coding), the author systematically coded interesting features of the data, before collating all codes into initial themes in phase 3 (generating initial themes). Phase 4 (developing and reviewing themes) involved writing short summaries of each theme and discussing them with a second author (JH) and patient representatives. During phase 5 (refining, defining, and naming themes), 1 author (AB-W) checked the themes against the raw data. Finally, in phase 6 (writing up), both authors selected vivid data extracts to illustrate each theme. Data from each stakeholder group (patients and practitioners) were initially analyzed separately. As the analysis progressed, we synthesized the accounts of patients and practitioners to triangulate the data [41].

### Reflexivity

Under a social constructivist approach, being reflexive is important, as the researchers are viewed as directly influencing the research process [37]. We engaged in reflexivity throughout this study, noting our reflections on the research process and critically questioning our own assumptions about therapeutic empathy [42]. In particular, we were mindful of beginning the research with our own definition of the concept, based on our previous research [11]. We were cautious of projecting the components identified through this earlier work onto participants’ accounts and consulted the raw data several times, along with the patient advisory board, to ensure that our analysis reflected participants’ views [42].

## Results

### Overview

In total, 16 participants (8 patients and 8 practitioners) took part in interviews lasting approximately 60 minutes each in June and July 2024. A total of 9 participants took part in interviews digitally, and 7 took part in person. Each participant was assigned a unique participant identification (ID) code; patients were assigned “PTCRX” to represent “patient or carer X,” and practitioners were assigned “HCPX” to represent “health care practitioner X.” Table 1 summarizes participants’ characteristics. In the interest of anonymity, patients’ specific experiences of health and care are not shared; collectively, they represent experiences of various cancers, disabilities, mental health conditions, and stroke.

Reflexive thematic analysis revealed considerable overlap between the views of patients and practitioners on what therapeutic empathy means and how it should be measured. Accordingly, the analysis generated three overarching themes that synthesize both groups’ perspectives. The themes included: (1) therapeutic empathy involves the practitioner showing the patient (that they are interested in the patient as a person, that they are actively listening, that they understand, that they are emotionally engaged, and that they are responding to their needs), (2) context matters, and (3) short, simple scales are a pragmatic approach to measurement. Given that the measurement of a concept is contingent on its definition [32], our analytic narrative considers the definition and measurement of therapeutic empathy together.

**Table 1.** Participant characteristics.

Participant ID code	Patient or practitioner (profession)	Age (years)	Sex	Ethnicity
PTCR1^a^	Patient	70	Female	White
PTCR2^b^	Patient	26	Female	Asian or Asian British
PTCR3	Patient and carer	64	Female	White
PTCR4	Patient and carer	59	Male	Asian or Asian British
PTCR5	Patient	55	Male	White
PTCR6	Patient and carer	65	Female	White
PTCR7	Patient	78	Male	White
PTCR8	Patient	48	Male	Asian or Asian British
HCP1	Practitioner (general practitioner)	54	Male	White
HCP2	Practitioner (palliative care consultant)	59	Female	White
HCP3	Practitioner (operating department practitioner)	35	Male	White
HCP4	Practitioner (radiographer)	37	Female	White
HCP5	Practitioner (midwife)	40	Female	White
HCP6	Practitioner (general practitioner)	45	Female	Asian or Asian British
HCP7	Practitioner (mental health nurse)	29	Female	White
HCP8	Practitioner (pharmacist)	56	Male	White

a PTCR: patient or carer.

b HCP: health care practitioner.

### Therapeutic Empathy Involves the Practitioner Showing the Patient

#### Overview

Participants defined therapeutic empathy as the empathy expressed by practitioners toward patients. They framed the concept as patient-centered, comprised of a number of interrelated attitudes and behaviors including showing an interest in the patient as a person, actively listening, demonstrating understanding, emotionally engaging, and responding to the patient’s needs. Importantly, participants emphasized that therapeutic empathy was about the practitioner successfully showing the patient that they were engaging in these attitudes and behaviors. For example, it was not enough for the practitioner to be interested in the patient as a person or to feel emotionally engaged; the practitioner also had to demonstrate this to the patient.

*I think practitioners need to bring a skill set, show that they understand us. It’s about acting like they care*.

[PTCR4]

*So, you know, it’s a clinician’s job to try and actually demonstrate empathy and show the patient they’ve heard and they care ... it ... needs to be communicated and demonstrated.*

[HCP1]

Accordingly, participants argued that any scale to measure therapeutic empathy should prioritize assessment of practitioner behavior. One practitioner stated “*...* what you really want to know is that the patient perceives those things that you’re experiencing and trying to communicate *...*” (HCP2).

#### That They Are Interested in the Patient as a Person

Showing an interest in the patient as a person was described as an important aspect of therapeutic empathy. Participants explained that this found expression in the practitioner asking open-ended questions about the patient’s familial, social, and cultural background. Relatedly, practitioners emphasized that the information obtained through such questions often provided important insights that could inform a patient’s care.

*... if you spend time asking questions about their actual lives, you’ll learn a lot ... you’re on the same wavelength ...*

[PTCR7]

*... what external things are happening in their life that are going to impact how they react ... and what they want from services? Ask them open-ended questions ... what’s their life like outside of hospital?*

[HCP5]

#### That They Are Actively Listening

Patients and practitioners considered active listening to be a core component of therapeutic empathy. They described this as the practitioner’s nonverbal communication, including eye contact, nodding, facing the patient, and making encouraging utterances. Practitioners added that they demonstrated active listening by repeating the patient’s words. Importantly, active listening was characterized as being nonjudgmental.

*I think they* [practitioners] *have to show they’re listening. And also, not jump to conclusions ...*

[PTCR6]

*... to show empathy, your body language should demonstrate active listening. That nodding ... showing that you are with them and listening to them ... it’s ... important*.

[HCP8]

#### That They Understand Their Perspective

Demonstrating an understanding of the patient’s concerns, emotions, and needs was perceived to be an essential part of therapeutic empathy. However, participants emphasized that it was impossible to fully understand another person’s thoughts and feelings. Instead, participants explained that practitioners could demonstrate understanding by sharing their own interpretation of the patient’s perspective, allowing the patient to confirm or refute their accuracy.

*I’ve noticed ... doctors ... when they are empathising, will say “it sounds like you feel anxious.” Or, “I can imagine that’s really hard for you.” And you think, “yeah, you’ve understood me*.”

[PCTR4]

*... demonstrating that you’ve got what they’re saying is either sharing what you hear ... or saying what you see ... sometimes we don’t get it right and it gives people a chance to correct us*.

[HCP2]

#### That They Are Emotionally Engaged

Participants took therapeutic empathy to involve the practitioner’s emotional engagement with the patient. They agreed that it was inevitable that the practitioner would feel something in response to their understanding of the patient’s perspective but emphasized that this feeling would not be the same as the patient’s. Participants struggled to precisely define the feelings they referred to, broadly describing practitioners as being “... moved emotionally ...” (HCP1). Verbally, practitioners might describe how they feel after hearing the patient’s story, while nonverbally, practitioners might have physical reactions to what they hear or see, such as holding a patient’s hand. However, participants emphasized that emotionally engaging, and demonstrating this, was a fine balance. Feeling and demonstrating too much emotion might risk the practitioner’s well-being, burden the patient, and impede the provision of care. Too little, and the patient-practitioner interaction may be perceived to be devoid of empathy.

*I think emotion, but to keep control of it, is important. I have been in situations when I’ve ended up therapeutically helping the person who’s supposed to be helping me. Which is too far the other way*.

[PTCR5]

*... emotion is part and parcel of the job ... there has to be a cut-off point ... where you’re not getting too invested into the patients’ lives so much that it affects your life ... otherwise you’ll be burnt out ...*

[HCP3]

#### That They Are Responding to Their Needs

Responding to the patient’s needs was described as an important part of therapeutic empathy. While both patients and practitioners acknowledged that responding to the patient’s needs may involve prescribing a medical treatment, they emphasized that the most helpful responses were often categorically “non-medical” (PTCR6), such as listening to the patient, validating their emotions, or offering small gestures.

*... one midwife ... gave me a massage because I was hurting ... we just talked ... she was showing that she cared, that she understood what I needed. It was the kind of thing a friend would do ...*

[PTCR1]

*... I suppose it’s responding to the patient’s individual needs ... sometimes you don’t have to do anything medical. I think sometimes people just want to be heard ...*

[HCP4]

### Context Influences Expression of Empathy

Participants identified contextual factors that influenced the expression of therapeutic empathy and that should be accounted for in the measurement of the concept. They explained that while all of the behaviors described earlier were important, not all would be appropriate in every context. For example, during an emergency, understanding and responding to the patient’s needs should take precedence over asking questions about their wider lives and emotionally engaging with them. In this context, instead of engaging in active questioning and listening, practitioners may prioritize obtaining a clinically informed understanding of the patient’s condition and taking action to help them. Several practitioners added that the expression of therapeutic empathy was dependent on time, particularly with complex patients. For example, in time-constrained consultations, exploring the patient’s wider familial, social, and cultural background may take up too much time. However, patients disagreed and argued that engaging in all of the behaviors associated with therapeutic empathy would likely save time long-term.

*... asking the patient questions about themselves and their lives ... that would save time further down the line ... you could push somebody down one route, and if you’d only asked the question, you probably wouldn’t pursue that course of action*.

[PTCR7]

*... you’re so pressured, aren’t you? And you have to be task focused and there are some questions that you have to ask ... as a clinician, you have to have an element of structure and move things on* ...

[HCP7]

Adding a further layer of complexity, participants stated that the expression of therapeutic empathy was influenced by the people within the context. They suggested that practitioners of different sexes, cultures, and ages may express therapeutic empathy differently. Similarly, participants thought that the patient’s characteristics would influence the expression of therapeutic empathy. For example, showing emotional engagement through physical touch might not always be appropriate.

*I mean, you’ve got the healthcare professionals, who because they understand ethnic minorities ... will greet my mother with a Hindu religious hello*.

[PTCR4]

*I think sometimes empathy can involve physical touch, putting a hand on patient ... but that’s straining to that area of what’s appropriate, what’s not appropriate ...*

[HCP1]

### Short, Simple Scales Are a Pragmatic Approach to Measurement

Participants identified scales as a pragmatic method of measuring therapeutic empathy because they could be distributed to a large number of people and offered flexibility around completion. Participants identified 4 possible versions of a therapeutic empathy scale: patient-reported, practitioner-reported, student-reported, and observer-reported. They argued that the patient-reported measure was most important because they perceived therapeutic empathy to be patient-centered. However, they acknowledged that there were limitations to all 4 proposed versions of a therapeutic empathy scale. Both practitioner-reported and student-reported scales were criticized for their subjectivity. A patient-reported measure was considered to be at risk of response bias because patients with negative experiences might be more motivated to complete it. An observer-reported measure was criticized for requiring trained raters to ensure its reliability. Acknowledging these limitations, participants proposed that measurement of therapeutic empathy should involve data from multiple sources and that all versions of a scale should correspond to allow comparison between groups.

*“... it’d be interesting to see how the patient scores the practitioner and how they score themselves ... you’d need to be able to match up those scores for either result to be meaningful.*

[PTCR3]

*I guess in terms of how you measure it ... for me it’s actually how it’s perceived by the patient because, you could be like really moved and not show that at all, and is that therapeutic empathy?*

[HCP1]

Although participants expressed a clear preference for using scales to measure therapeutic empathy, this was contingent on the scales being short and simple. Short scales were considered essential for time-poor practitioners and for patients who were unlikely to want to spend a long time assessing their practitioner’s empathy. Several participants specifically suggested a maximum of 5 items. Simplicity was perceived as facilitating shortness of scale length and as being necessary for ensuring accessibility across different populations if all versions of the scale were to correspond. Participants proposed that simplicity could be achieved by using lay language that would minimize the possibility of misinterpretation and, in turn, inaccurate responses.

*I think the more questions, the more they’ll be like “oh God, another long questionnaire” and not finish it ... people tend to like shorter questionnaires*.

[PTCR2]

*I’d be prepared to spend a couple of minutes completing a scale ... you lose the essence of it if you break it down too much. And, for patients, lots of questions can be daunting ... they might not understand the difference between the questions ...*

[HCP2]

## Discussion

### Summary of Findings

This study has, for the first time, synthesized the perspectives of patients (with diverse lived experiences of health and care) with practitioners (from different professions) on what therapeutic empathy means and how it should be measured. The findings show that patients and practitioners define therapeutic empathy similarly as involving practitioners demonstrating 5 attitudes and behaviors: an interest in the patient as a person, active listening, understanding, emotionally engaging, and responding to the patient’s needs. Expression of these attitudes and behaviors may depend on contextual factors, including the clinical scenario, time, and the patient themselves. Both patients and practitioners favored short, simple scales assessing all 5 attitudes and behaviors as an approach to measuring therapeutic empathy.

### Comparison With Other Evidence

A recent review and thematic analysis of existing therapeutic empathy definitions identified 6 components, including exploring, understanding, shared understanding, feeling, therapeutic action, and maintaining boundaries [11]. These components align well with the attitudes and behaviors identified by participants in this study. “Exploring” is similar to showing an interest in the patient as a person and actively listening. “Understanding” and “shared understanding” map on to participants’ description of understanding. “Feeling” and “maintaining boundaries” share similarities with the subtheme emotional engagement, and “therapeutic action” corresponds with responding to the patient’s needs.

Our findings also share similarities with previous studies [14-16] of patients’ experiences of therapeutic empathy. These studies identified listening, understanding (the patient as a whole person, including their emotions), and taking action to help the patient as characteristics of therapeutic empathy [14-16]. While these findings align with ours, we also found that the practitioner’s emotional engagement, including their own feelings in response to the patient’s situation, was perceived to be an important part of therapeutic empathy to patients and practitioners. This finding sits partly at odds with previous research, which found that practitioners rate emotional involvement as more empathic than patients [17].

This study offers 2 important additions to the evidence base. First, the findings suggest that not all of the elements of therapeutic empathy might be appropriate in all contexts for all patients. Participants identified contextual factors that necessitated the foregrounding of particular empathic behaviors over others. Second, participants implied that therapeutic empathy must be successfully demonstrated to patients in order to be meaningful. Previous definitions of therapeutic empathy have focused on the practitioner’s experience of empathy (eg, how they understand or feel), with limited consideration given to how this is conveyed to the patient [11]. Our findings emphasize that the latter is what makes empathy therapeutic. However, further research is needed to determine how to best express therapeutic empathy to patients in practice, before concrete recommendations or strategies can be suggested regarding specific behaviors to teach or adopt.

That being said, there are evidence-based strategies to convey empathy that align with the themes generated in this study [43]. For example, looking at the patient when talking to them (instead of a screen or paper notes), avoiding interrupting the patient, and being genuinely curious about them [43] align strongly with our themes pertaining to showing genuine interest in the patient and actively listening to them. Similarly, using facial expressions and other nonverbal communication to show understanding largely reflects our theme about understanding the patient’s perspective, while giving positive messages of hope shares similarities with the theme about responding to patients’ needs [43].

Relatedly, our findings clearly emphasize practitioners demonstrating empathic behaviors toward patients. This contrasts with previous research that contests that empathy is an innate “trait,” something a practitioner either has or does not have [44-46]. As such, our findings contribute to the “state or trait” debate surrounding therapeutic empathy, introducing the possibility that a practitioner with limited “trait” empathy could, in theory, be perceived as empathic by their patients through the successful enactment of empathic behaviors.

Our findings also add to the debate over whether therapeutic empathy is an affective or cognitive concept. Affective empathy has long been disregarded by the clinical community for fears that it would hamper objectivity and lead to burnout [9,47]. More recently, however, authors have begun to argue that therapeutic empathy includes cognitive and affective components [1,48]. The findings of our study align with the latter argument, showing that patients’ and practitioners’ conceptualizations of therapeutic empathy include both cognitive and affective aspects.

Many existing measures of therapeutic empathy are long [22-29,34] and include negatively worded items [24,26,28,29,31]. This leads to potential problems with the reliability and validity of the responses generated [33]. This may be, in part, due to the fact that the development of existing measures has seldom included the patient voice [15,32]. This study revealed that both patients and practitioners have a preference for short, simple scales to measure therapeutic empathy. Moreover, our findings additionally highlight the absence of and need for corresponding patient-, practitioner-, student-, and observer-reported measures to allow triangulation of scores. Development of such a scale should be a priority for further research, given that there are discrepancies between practitioner self-ratings of empathy and patient ratings of practitioner empathy [19-21] that may prevent patients and practitioners from benefiting from therapeutic empathy downstream [4-7].

Finally, our finding that what patients consider to be empathic care varies according to context dovetails with the literature showing that what patients take to be empathic care is relative to ethnicity and culture [49].

### Strengths and Limitations

Unlike previous research [13-17], this study explores and synthesizes patients’ (with diverse experiences of health and care) with practitioners’ (from different professions and health care services) perspectives on the definition of therapeutic empathy. Moreover, this is the first study to explore how therapeutic empathy should be measured from the perspectives of the stakeholders who will complete such measures. However, despite being conducted rigorously, this study has potential limitations. First, the sample was comprised of patients and practitioners from a medical school and a school of health care from 1 institution. As such, participants may not be representative of all patients and practitioners, and their views may have been, at least in part, shaped by their experiences at that institution. This is mitigated somewhat by our purposeful sampling of patients with different experiences of health and care and different ages, sexes, and ethnicities, along with practitioners from different professions and health care services and different ages, sexes, and ethnicities. Moreover, the purpose of this study (and indeed the purpose of most qualitative research) [37] was not to recruit a sample representative of all patients and practitioners, but rather to explore, in depth, diverse patients’ and practitioners’ views on therapeutic empathy. Additionally, we have provided a rich description of the context in which this research took place, supporting reflection about the transferability of the findings to other contexts [37]. Another potential limitation is that, to accommodate participants’ needs, we offered them the option of completing their interviews digitally, and 9 participants opted for this. In-person interviews are considered the gold standard in qualitative data collection [50]; however, research shows that the depth and length of the data generated from web-based interviews are similar [51,52].

### Recommendations for Further Research

Further research is needed to explore the ways by which the attitudes and behaviors that comprise therapeutic empathy are enacted in clinical practice and how this varies across different contexts (including those influenced by ethnic, cultural, and systemic factors), clinical scenarios (particularly those that may be considered challenging, like breaking bad news), and patient and practitioner demographics. This might be achieved using conversation analysis of video-recorded consultations. Relatedly, exploring perspectives on empathic communication beyond patient-practitioner interactions—for example, intra- and interprofessional empathy—and how this shapes patients’ perceptions of therapeutic empathy in practice would be a worthwhile avenue for further research. Moreover, research would benefit from replicating this study with a larger sample across multiple contexts to develop our findings further. Finally, research should be conducted to develop and psychometrically test a “universal” therapeutic empathy measure that can be completed by patients, practitioners, students, and observers and is sensitive to context and complexity.

### Conclusions

Little previous research has explored the synergies between views of patients and practitioners on the definition and measurement of therapeutic empathy and those who do often emphasize the differences between their perspectives [13,17]. On the contrary, we found that patients and practitioners have similar views on what therapeutic empathy means and describe it as involving the practitioner demonstrating 5 attitudes and behaviors to patients. These include showing an interest in the patient as a person, actively listening, understanding, emotionally engaging, and responding to the patient’s needs. This perspective innovatively positions therapeutic empathy as a professional behavior and skill that should be demonstrated irrespective of patient reciprocity. A novel finding is that contextual factors, including the clinical scenario, time, and the patient, may influence whether, and how, these empathic attitudes and behaviors are expressed in practice. Measures of the concept should be developed with consideration for the role of context in empathy expression but, importantly, should be short and simple, unlike many existing measures.

## Supplementary material

10.2196/71610 Multimedia Appendix 1 Semistructured interview topic guide.

## Abbreviations

HCP: health care practitioner
PTCR: patient or carer
