# Supplementary material for: Patient and Practitioner Perspectives on the Definition and Measurement of Therapeutic Empathy: Qualitative Study
Source: J Particip Med. 2025 Jul 31;17:e71610. doi: 10.2196/71610 (PMC12313346; doi:10.2196/71610)
Supplement: Multimedia Appendix 1 [file jopm-v17-e71610-s001.docx]

**Patient and practitioner perspectives on the definition, practice, and measurement of therapeutic empathy**

**Topic guide**

*Topic guide is designed to be iterative and evolve as the research progresses and evolves.*

**Briefing (pre-interview/focus group):**

- Ensure setting is conducive with quiet and focussed conversation (whether in person or online).
- Test audio recorder.
- Welcome participant and explain what the discussion will entail:

“I am going to be asking you some questions about your views and experiences of empathy in healthcare. I have a list of questions to guide our discussion, but we don’t need to follow these strictly – they are just there to help me. There are no right or wrong answers and I want you to feel that you can be open and honest about your experiences.”

- Hand out participant information sheet and consent form, ask for any questions or needed clarifications.
- Collect a signed consent form.
- Begin audio recording, stating who is being recorded, date and time.

1. **How would you define empathy in healthcare?**
   1. What do you think empathy means?
   2. What does empathy in healthcare involve?
   3. What should empathy in healthcare *not* involve?
   4. Why is empathy in healthcare important?
2. **Can you tell me about any memorable experiences you have of empathy in healthcare?**
   1. What made this memorable?
   2. What made it a good/poor example of empathy?
3. **How do you think empathy is enacted in healthcare?**
   1. What is the practitioners’ role?
   2. What is the patients’ role?
4. **What behaviours demonstrate empathy?**
   1. What should practitioners do to show empathy for their patients?
   2. Does empathy require the patient to behave in a certain way?
5. **We have done some research and developed our own definition of empathy in healthcare, which we call therapeutic empathy. Our definition states that “Therapeutic empathy involves *exploring* and *understanding* the patient’s perspective*,* reaching a *shared understanding, feeling* in response to *understanding,* and taking *therapeutic action,* while *maintaining boundaries.”*How does our definition fit with your understanding of empathy?**
   1. What do you think the different components mean?
   2. Which components do you think are most important?
   3. How do you think each of the components can be expressed in practice?
   4. Which parts do you agree with?
   5. Are there parts that you disagree with?
   6. Are there components of empathy that you think we have missed?
6. **Practitioners’ empathy levels are usually assessed using questionnaires, which are either self-assessed (where practitioners answer questions about their own empathy) or patient-assessed (where patients answer questions about their practitioner’s empathy). What do you think about this?**
   1. What do you think the advantages are of measuring empathy using questionnaires?
   2. What do you think the disadvantages are of measuring empathy using questionnaires?
7. **With this in mind, how do you think therapeutic empathy would best be measured?**
8. **What features do you think a measure of your own/your practitioner’s empathy levels should include?**
   1. How long should it take to complete?
   2. How many questions should it include?
   3. How could we make the questionnaire user-friendly?
   4. What sort of things would you expect the questions to assess? (e.g., attitudes or behaviours?)
9. **Is there anything that we haven’t covered today that you would like to add?**

**De-brief (post-interview/focus group)**

- End recording.
- Thank participant for taking part.
- Ask them if they have any questions/concerns that would like to be addressed.
- Explain that we will share the results with them at the end of the study.
